# Impressive pan-genomic diversity of *E. coli* from a wild animal community near urban development reflects human impacts

**DOI:** 10.1016/j.isci.2024.109072

**Published:** 2024-02-01

**Authors:** Katherine M. Lagerstrom, Nicholas C. Scales, Elizabeth A. Hadly

**Affiliations:** 1 Department of Biology, Stanford University, Stanford, CA, USA; 2 Department of Ecology and Evolutionary Biology, University of California, Irvine, Irvine, CA, USA; 3 Stanford Woods Institute for the Environment, Stanford University, Stanford, CA, USA; 4 Center for Innovation in Global Health, Stanford University, Stanford, CA, USA; 5 Department of Earth Systems Science, Stanford University, Stanford, CA, USA

**Keywords:** Microbiology, Wildlife microbiology

## Abstract

Human and domesticated animal waste infiltrates global freshwater, terrestrial, and marine environments, widely disseminating fecal microbes, antibiotics, and other chemical pollutants. Emerging evidence suggests that guts of wild animals are being invaded by our microbes, including *Escherichia coli*, which face anthropogenic selective pressures to gain antimicrobial resistance (AMR) and increase virulence. However, wild animal sources remain starkly under-represented among genomic sequence repositories. We sequenced whole genomes of 145 *E. coli* isolates from 55 wild and 13 domestic animal fecal samples, averaging 2 (ranging 1–7) isolates per sample, on a preserve imbedded in a human-dominated landscape in California Bay Area, USA, to assess AMR, virulence, and pan-genomic diversity. With single nucleotide polymorphism analyses we predict potential transmission routes. We illustrate the usefulness of *E. coli* to aid our understanding of and ability to surveil the emergence of zoonotic pathogens created by the mixing of human and wild bacteria in the environment.

## Highlights

• Wild animals host a large diversity of *E. coli* including strains of human origin
• Individuals carry multiple strains simultaneously, facilitating within-host mixing
• Clinically relevant antimicrobial resistance was found on mobile genetic elements
• Transmission of *E. coli* occurs in the wild and across the wildland-urban interface

## Graphical abstract

**Figure:**
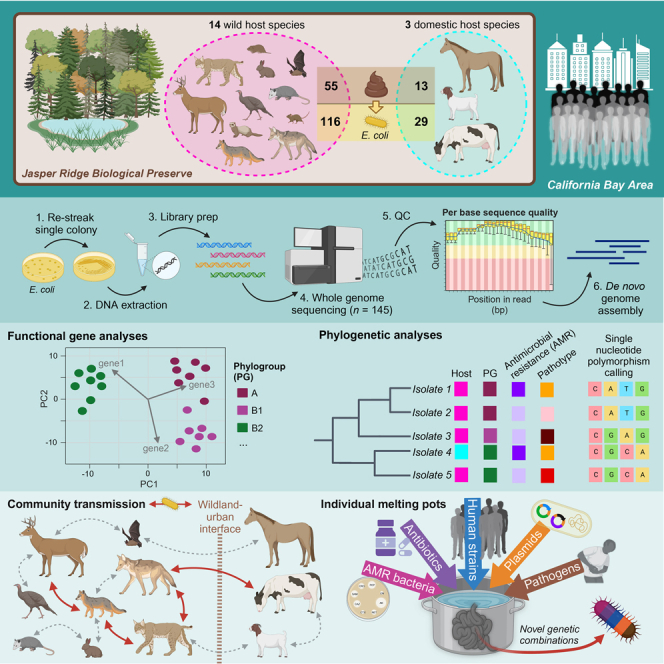

## Introduction

### Major gaps in wild animal studies

Despite its fame (or infamy), our understanding of the life of *Escherichia coli* that exists outside of human and domesticated animal guts remains in its infancy.^1^ Of the few investigations of *E. coli* in wild animals, surprising diversity has been revealed,^2^ increasingly with genes conferring clinically important antimicrobial resistance (AMR).^3^ While *E. coli* diversity is under-sampled in wild animals,^1^ it has also been obscured by selective plating methods, where stool samples are directly subjected to antibiotics before typing, thereby eliminating substantial non-resistant diversity, or even potentially creating resistances during the laboratory methods.^4^ Shallow sampling methods within and between individuals are also to blame, as the recurrent presumption that a single isolate per host is representative of the within-host *E. coli* diversity overlooks substantial within-individual and within-host species diversity.^5^ The lack of genomic studies on *E. coli* from wild animals is critical, especially as wild animals may serve as reservoirs of harmful bacteria or even “melting pots” that could facilitate novel genetic combinations of virulence and AMR genes that could threaten global public health.

### Problems with pathogenic *E. coli*

Two major categories of pathogenesis have been defined in *E. coli*: intra-intestinal pathogenic *E. coli* (InPEC) or diarrheagenic *E. coli*, which include the well-known Shiga-toxigenic *E. coli* (STEC); and extraintestinal pathogenic *E. coli* (ExPEC), which causes infections outside of the gut and is responsible for the death of over 2 million people per year.^6^ ExPEC strains include uropathogenic *E. coli* (UPEC), the leading cause of urinary tract infections (UTIs), and meningitis-associated *E. coli* (NMEC), which can cause bloodstream infections and is the most common cause of neonatal meningitis.^7^ Just 7 foodborne pathogens, one of which is *E. coli*, have been estimated to cost the United States anywhere between US$ 6.5 billion and US$ 35 billion annually.^8^ Though there is no evidence that wild animals are a significant or repeated source of food contamination by pathogenic *E. coli*, there are occasional reports of wild animals contaminating people or their food sources.^9^^,^^10^ In response to fear, farmers often resort to environmentally detrimental methods of wildlife exclusion, including trap-and-kill and removal of non-crop vegetation surrounding croplands.^11^ It is exceptionally challenging to source trace when outbreaks do occur, in part due to limitations of the *E. coli* genome database, but also because *E. coli* is notoriously genetically diverse. From genomic studies on isolates from humans, the core genome, or those genes found in every isolate of the species *E. coli*, has been estimated to be just 6% of the pan-genome, *i.e*., the total gene pool encompassed by all *E. coli*.^12^ The pan-genome of *E. coli* could be nearly infinite, attributed in great part to horizontal gene transfer (HGT) between strains and other bacterial species.^13^

### Compounding impacts in the Anthropocene

The need to deepen our understanding of *E. coli* is most acute at the human-domestic-wildlife interface where spillover and spillback occur.^14^ Evidence suggests that *E. coli* is readily propagated by the movement of hosts across geographic space, particularly migratory birds,^15^^,^^16^^,^^17^^,^^18^^,^^19^^,^^20^ but is also facilitated by human activity and environmental factors.^21^ Human encroachment into previously wild areas is a hallmark of the Anthropocene, and human-animal encounters happen frequently in both agricultural and highly populated areas. Increasingly smaller nature areas lead diverse wildlife to interact more frequently, further contributing to the sharing of potential pathogens.^14^ Strain-sharing of *E. coli* has been documented between humans and domestic animals,^22^ between livestock and sympatric wildlife,^23^^,^^24^ and between humans and wild animals proximal to urban development.^25^^,^^26^ Climate change and biodiversity loss further drive rates of cross-species transmission and thus the emergence of infectious diseases.^14^^,^^27^ Extreme weather often compromises water quality, like droughts, which concentrate microbes in water, and extreme rainfall events, which distribute them. Indeed,the prevalence of diarrheal disease outbreaks attributed to *E. coli* is significantly higher following intense rain events and subsequent flood pulses than during the dry season, a trend likely to worsen as climate change is predicted to intensify the variability and frequency of extreme weather events.^28^

To make matters worse, due to the extensive use of antibiotics and other antimicrobials in human and veterinary medicine, and in animal and plant agriculture, such chemicals are increasingly infiltrating the environment. Today, the World Health Organization identifies AMR as one of the biggest threats to global health, development, and security.^29^ The spread of antibiotics and AMR bacteria into the environment is facilitated by livestock and agricultural runoff. With more episodic and intense rainfall, the risk of wastewater lagoons breaching at concentrated animal feeding facilities and urban water treatment plants will increase, resulting in more runoff migrating into groundwater supplies, nearby agricultural lands, and marine environments. Urban wastewater treatment processes may also contribute to concentrating and increasing the abundance of AMR by inefficient drug removal and the large-scale mixing of bacteria in the treatment process. For example, a recent study showed the abundance of antimicrobial resistance genes (ARGs) was significantly higher in sewage treatment plant effluent than its influent.^30^ Such extensive environmental pollution by antibiotics has created unprecedented selective pressures on bacteria and contributes to the rapid and global spread of resistance.^31^ *Escherichia coli* plays an important role in the global AMR crisis; not only for its propagation of ARGs via HGT, but *E. coli* itself is among the leading causes of mortality associated with drug-resistant infections. It alone was responsible for the most deaths attributed to AMR in 2019, an estimated 800,000 of a total of 4.95 million globally.^32^ Compounding this already urgent issue, the presence of other agrichemicals in the environment, such as Roundup®, increases the mutation rate in *E. coli* and results in more rapid development of AMR, in some cases, as much as 100,000 times faster.^33^ Increasingly, research indicates that wildlife harbors a non-trivial level of clinically relevant AMR *E. coli*, and evidence strongly suggests anthropogenic sources.^3^^,^^34^^,^^35^^,^^36^

### Research objectives

We investigated *E. coli* genomes from wild and domestic animals in an interacting community imbedded in an urban landscape to (1) capture the genetic and functional diversity of *E. coli* both among the community and within an individual, (2) assess strain-sharing between hosts to predict how *E. coli* might move through this network, (3) evaluate the prevalence of pathogenic (to humans) *E. coli*, AMR *E. coli*, and human-associated sequence types (STs) in domestic and wild animals, and (4) consider the potential for the guts of wild animals to act as melting pots of novel genetic combinations that could threaten public health. We hypothesize that the genetic repertoire of wild animal *E. coli* will reflect the presence of domestic animals, as well as historical and present human impacts on the preserve and its waterways, and that wild animal species are differentially impacted depending on their ecology and life-history attributes.

## Results

### Phylogenetic and pan-genomic analyses

**Figure 1:**
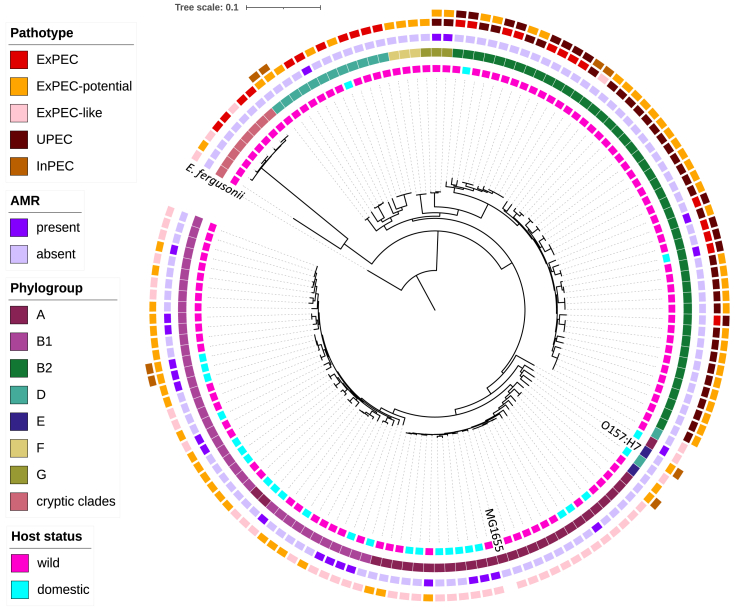
Genetic and ecological structure of *E. coli* from JRBP RECOPHY phylogeny of 143 *de novo* constructed *E. coli* genomes from animals at JRBP and including reference genomes MG1655 and O157:H7, rooted by *E. fergusonii*. Colored strips from inside out indicate host status*, E. coli* phylogroup, AMR, and pathotype (some isolates met criteria for multiple pathotype assignments). Graphic created in iTOL v5.

Eight major phylogroups of *E. coli* have been described, each associated with distinct characteristics and ecological niches.^37^ The original Jasper Ridge Biological Preserve (JRBP) *E. coli* collection encompassed all major phylogroups, determined by a PCR-based phylogrouping method,^5^ however, we did not successfully sequence an isolate belonging to phylogroup C. After removing 2 genomes that did not pass quality control, the resulting phylogeny of 143 *de novo* constructed *E. coli* genomes from wild and domestic animals at JRBP showed a broad distribution of genotypes across host status, AMR, and pathotype categories, and mostly agreed with the mono-phyletic groupings, with a few exceptions (Figure 1). Some mixing was observed between phylogroups D and E, suggesting potential mutation in the phylogroup-specific gene primers used in ClermonTyper to assign strains to phylogroups based on the differential presence of a few select genes. Three isolates of phylogroup A, all from horses, did not cluster with the rest of phylogroup A, potentially for the same reason.

**Figure 2:**
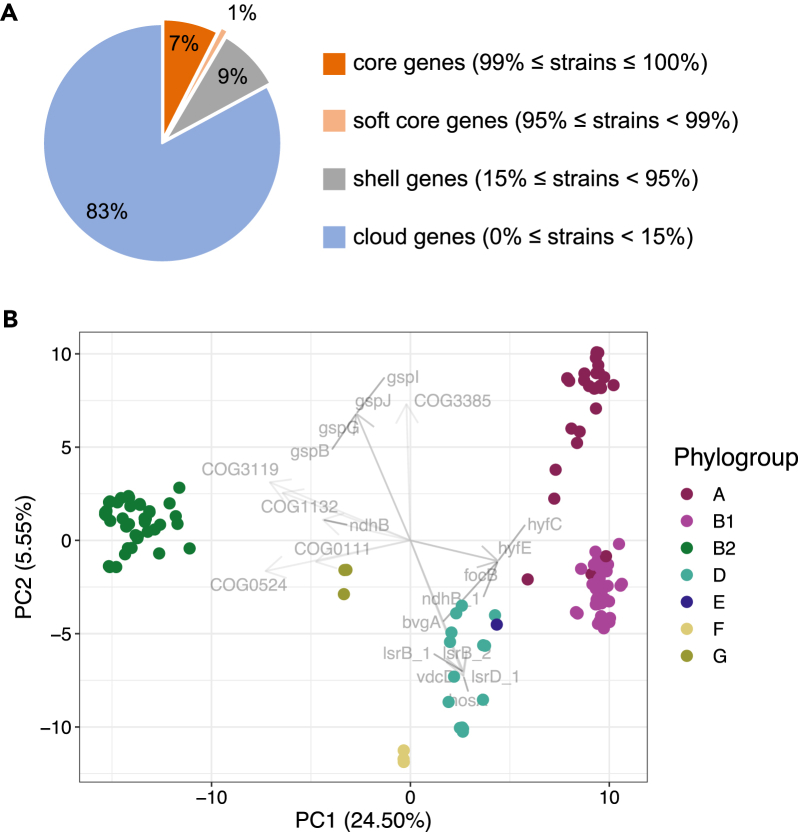
Gene and functional diversity of *E. coli* from JRBP (A) Percentages of gene families out of the total pan-genome belonging to the core and accessory (shell and cloud) genomes. (B) *E. coli* phylogroup functional similarity using principal component analysis (PCA) of 135 *E. coli* genomes (excluding cryptic clade isolates; n = 8). Each dot corresponds to a genome in the first 2 principal components (PC). Vectors show the top 5 annotations with the largest PC1 and PC2 values in both positive and negative directions.

Pan-genome analysis of 143 *E. coli* whole genomes from JRBP identified 32,028 gene families (over 7 times the average genome size), only 8% of which were shared among > 95% of the isolates. Just 17% of those were shared between > 15% isolates (Figure 2A). In total, 53 *fimH* types, 92 serotypes (O:H antigens), and 88 sequence types (STs; assigned by Achtman 7 gene MLST) were represented. Nine of these STs were assigned sequentially in EnteroBase (ST13010 through ST13018), indicating that these were novel to the database.

Principal component analysis (PCA) of the count matrix combining all PROKKA assignments (to a gene name, COG, or EC number) resulted in a PC1 axis that explained 24.5% of the total variance and phylogroup B2 being the most distinctly separated from other phylogroups (Figure 2B). Phylogroups A and B1 primarily separate in PC2, suggesting a more similar gene repertoire, while phylogroup E nests within phylogroup D. Interestingly, *Escherichia* isolates belonging to the cryptic clades separate clearly from the phylogroups, suggesting that they harbor a unique gene repertoire that distinguishes them, thus supporting their distinction from *E. coli sensu stricto* (Figure S1). Functional analyses were severely limited as 46% of genes in the pan-genome were unassigned to a functional COG (cluster of orthologous genes) category (Figure S2).

### Within-scat diversity supports melting pot hypothesis

We sampled more than 1 *E. coli* isolate (averaging 3) from the same scat for 41 different individual samples, capturing substantial within-host genetic diversity. Though we did not sample each scat to saturation at the phylogroup or ST level, which would have involved constructing rarefaction curves and sampling additional isolates until the discovery of new diversity was exhausted, we still found that 28 of those individual scats sampled to n > 1 isolates carried > 1 phylogroup. Moreover, 29 scats carried > 1 ST, 13 of which also carried different STs of the same phylogroup (Table S1). Notably, 4 different STs were sequenced from each of a single opossum, puma, turkey, coyote, deer, and horse scat, as well as the goat community. This suggests that with-host diversity could be far greater, as these results were limited by the number of isolates sequenced per scat sample.

### Possible recent transmission events

**Figure 3:**
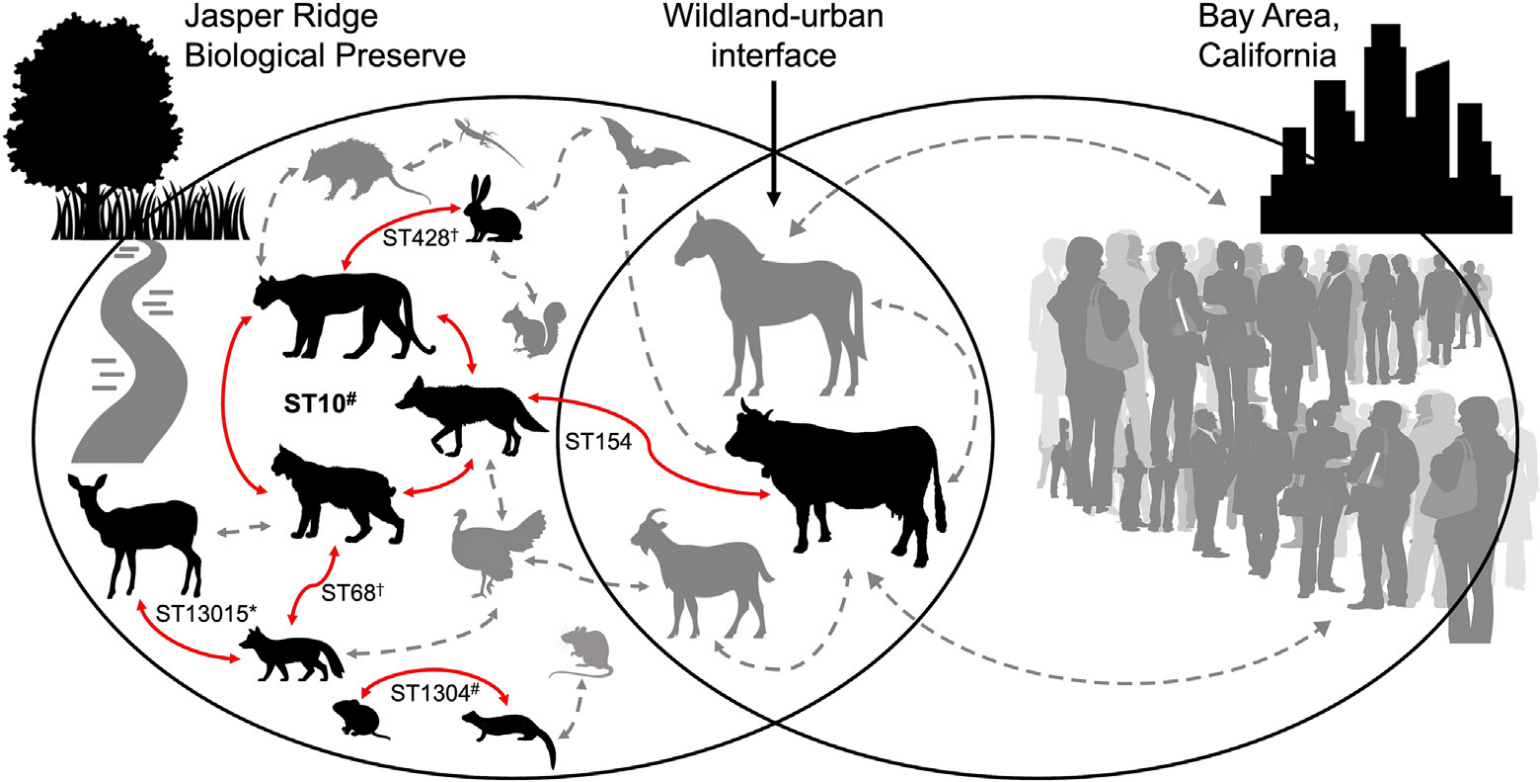
Possible recent transmission at JRBP Red arrows indicate potential clonal relationships identified among *E. coli* from different host species as defined by ≤ 10 SNP differences between pairs of isolates. The sequence types (STs) of those isolates are indicated. Gray arrows suggest that transmission is occurring throughout the JRBP ecosystem and across the WUI. ^**#**^Same collection day; ^∗^Likely a novel ST; ^**†**^ExPEC.

Both our sampling methods and the proximity of animals in this community allowed us to capture 62 potential clonal relationships between pairs of isolates as defined by the observation of ≤ 100 single nucleotide polymorphisms (SNP) between genomes. Thirty-six of these were pairs of isolates taken from the same scat sample and 7 pairs were derived from different scat samples but from the same host species. Due to the opportunistic nature of our sample collections, we were unable to confirm whether these samples came from the same or different individual(s). So, this could either indicate clonal relationships of *E. coli* occurring between different individuals of the same host species, or persistence of a clone within the gut of an individual for at least the amount of time that occurred between sample collections. However, 19 potential clonal pairs were derived from scat samples of different host species, so for these, we were certain they came from different individuals. Fourteen of those 19 pairs had ≤ 10 SNP differences between them, a conservative measure to indicate a probable strain-sharing event. These 14 clonal pairs represented 6 STs shared between 13 individuals. ST10 clones were isolated from each of a puma, a bobcat, and a coyote, all 3 scat samples from which were collected on the same day (Figure 3). A potential clonal pair of ST1304 isolates from a vole and a weasel were also collected on the same day. Notably, 2 potential clonal relationships were of ExPEC strains, and 1 crossed the wildland-urban interface (WUI). Table S2 summarizes the isolate metadata, SNP rate, and SNP count for all 62 pairs of strains identified with potential clonal relationships.

### Virulence factor profiles

The number of distinct virulence factors (VFs) per genome in the JRBP *E. coli* isolates ranged from 11 to 46 (Table S3), meaning zero isolates qualified as “low pathogenicity” by our criteria (Table S4). The prevalence of ExPEC strains was 41.3%, and of InPEC strains, 4.9% (Table S5). No isolates were positive for the gene *bfpA* (bundle-forming pilus), so those containing the gene coding for the enteropathogenic *E. coli*-associated intimin protein, *eae,* were considered atypical; aEPEC.^38^ Of the wild hosts, at least one pathogen was obtained from 65.3% of individuals, as well as the squirrel midden and bat roost. Of the domestic hosts, a pathogen was found in 18.2% of individuals (just 2 horses), and the goat herd.

All phylogroup B2 isolates qualified as pathogens (ExPEC or InPEC) by our classification criteria, followed by phylogroup G (66.7%), phylogroup D (64.3%), and the cryptic clades (50%), though the sample sizes for phylogroup G and the cryptic clades were low (Table S5). Conversely, no ExPEC or InPEC isolates belonged to phylogroups E or F, and phylogroups A and B1 had less than 10% pathogen prevalence (Table S5). Principal component analyse (PCA) on the count matrix of VFs per genome showed phylogroup B2 separating from all other phylogroups with most top PC loadings associated with the direction of the phylogroup B2 cluster. However, *lpfA* (long polar fimbrial subunit A), associated with mobility, was identified in the top loadings in the direction of all other phylogroups (Figure S3).

### Antimicrobial resistance gene profiles

In total, 16.1% of all isolates carried at least one antimicrobial resistance gene (ARG); 70.6% of those were multi-drug resistant (MDR), defined by the presence of > 2 ARGs. These isolates derived from 17 scat samples of 7 host species: bobcat (57.1% of scats tested), coyote (60.0%), gray fox (16.7%), opossum (100%), turkey (25.0%), horse (42.9%), and the goat herd. This represents 23.6% of all wild hosts and 33.3% of all domestic hosts, or 28.6% of domestic-sourced isolates and 13% of wild-sourced isolates. Remarkably, all AMR isolates from both horses and coyotes were MDR. Phylogroup G (66.7%), followed by B1 (34.2%), contained the most AMR isolates (Table S5). Collection sites of scat samples with AMR were concentrated on the horse trail along the northern border of the preserve and on the most heavily foot-trafficked trail encircling the reservoir (Figure S4).

**Figure 4:**
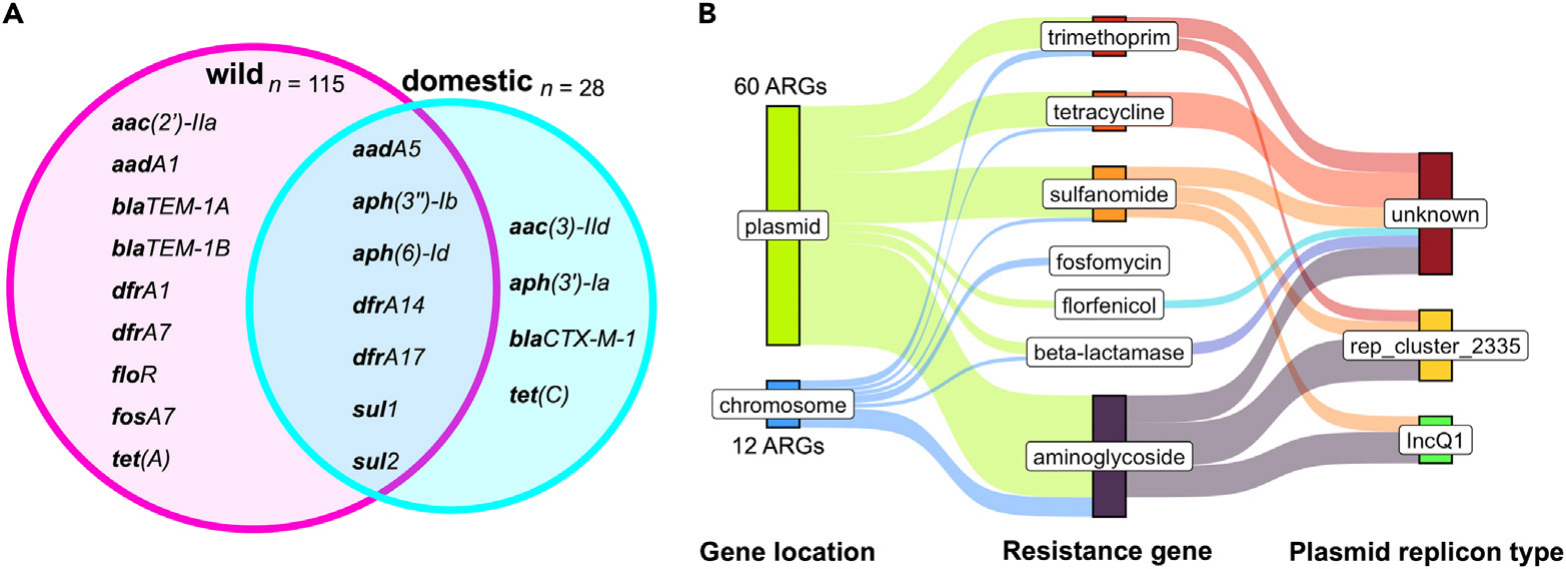
Ecological and genomic contexts of AMR at JRBP (A) Of 20 unique ARGs identified by ResFinder among *E. coli* genomes from JRBP, 9 were unique to isolates from wild animals, 4 were unique to isolates from domestic animals, and 7 were shared between wild and domestic animals; *n* = number of genomes per group. (B) Sankey diagram detailing the genomic locations (plasmid vs. chromosome) of all 72 ARGs identified across 143 genomes linked to their predicted resistance phenotype and when plasmid-based, their plasmid replicon type.

Of 72 total ARGs identified among JRBP *E. coli* isolates, 20 were distinct, with 9 exclusively found in wild animals, 4 exclusively in domestic animals, and 7 (35%) shared between domestic and wild animals (Figure 4A). Furthermore, 84% of ARGs were plasmid-based, representing 6 classes of commonly prescribed antibiotics (Figure 4B). Fosfomycin resistance was the only ARG found solely in the chromosome. The plasmid replicon types of the majority of plasmid-based ARGs were unknown to the database (n = 30), but plasmid IncQ1 was associated with 12 ARGs and plasmid rep_cluster_2335 was associated with 18 ARGs (Table S6). The latter assignment to a non-descript replicon type ("rep cluster") indicates that the plasmid has been identified previously, but is poorly researched to-date.

### Prevalence of human-associated sequence types

A handful of STs belonging to phylogroups B2 and D are responsible for the majority of ExPEC infections and are largely human-associated. In phylogroup B2, these are ST73, ST95, and ST131, and in phylogroup D, ST69.^39^ ST131 is the most prevalent ExPEC clone, mainly colonizing humans and human-associated animals,^40^ and was identified in the bat roost near JRBP. This isolate belonged to clade B (O25:H4, *fimH*22) and classified as a UPEC. PlasmidFinder suggested that it may carry up to 3 plasmids (IncFII, IncXI, and pEC4115), but no ARGs were identified, suggesting that it is related to, but not the same MDR clone that has gained much recognition for its rapid global dissemination in the past decade.^41^ ST73, isolated here from a domestic horse, has previously been identified in companion animals^39^ and has been shown to be shared between canine and human members of a household experiencing UTIs.^42^ A predominant cause of trimethoprim-sulfamethoxazole-resistant UTIs across the United States,^43^ *E. coli* ST69 was isolated once from each of 3 different host species: puma, coyote, and opossum. The ST69 isolate from a coyote also carried 7 ARGs. Other important human-associated STs identified in wild animals at the preserve include ST127, isolated from an opossum, which is over-represented in human pneumonia isolates^44^ and has been described as “a recently emerged global pathogen,”^45^ ST117, isolated from 2 coyotes, one which also a UPEC with 3 ARGs, and ST155^46^ also from a coyote and carrying 3 ARGs.

### Diversity within ST10

**Figure 5:**
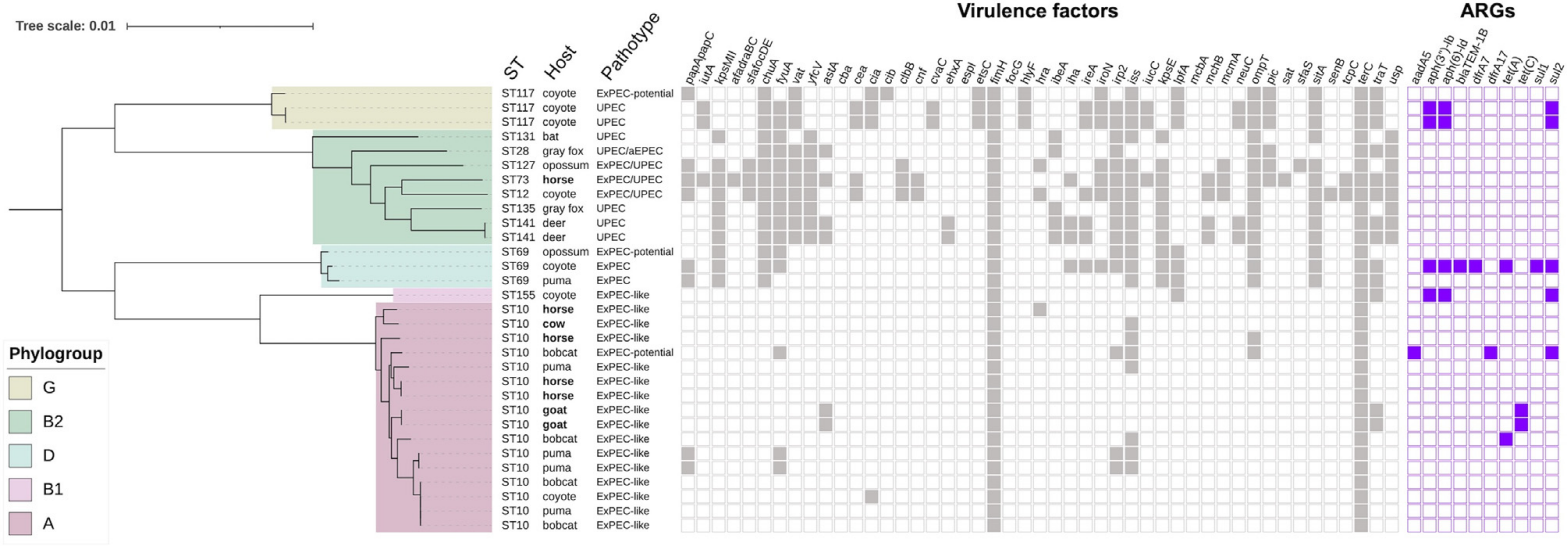
Phylogenetic and metadata analysis of human-associated STs at JRBP A maximum-likelihood tree was based on RAxML-NG under GTR+GAMMA with default parameters on the core gene alignment (Roary) of 31 human-associated STs. Presence or absence of the 5 ExPEC-associated VFs, 4 UPEC-associated VFs, and the 38 other major VFs (gray) and ARGs (purple) are detailed for each isolate. Domestic hosts are shown in bold.

The most common ST at JRBP was ST10, comprising 11.2% of all isolates. Substantial genetic diversity was observed within these 16 isolates, encompassing a pan-genome themselves of 7,080 gene families, just 51.5% shared among > 95% of isolates, representing 10 distinct serotypes and 5 different *fimH* types. This is evidence corroborating previous findings that ST10 has huge antigenic diversity and greater genetic diversity overall than other STs assessed.^47^ ST10 was present in all domestic host species, but in only 3 of 14 wild species (puma, bobcat, and coyote). PCA on the count matrix of genes in only the ST10 isolates showed substantial overlap in the gene repertoires of isolates from wild and domestic animals, however greater functional gene diversity was encompassed by those from wild animals. This could suggest potential strain-sharing between domestic and wild animals on the preserve (Figure S5). Including those listed above, human-associated STs composed 21.7% of all JRBP isolates, but their prevalence was higher among domestic hosts (28.6%) than wild hosts (20.0%). Controlling for some individual hosts contributing multiple isolates, 50% of domestic hosts and 36.4% of wild hosts carried at least 1 of these human-associated STs. The phylogenetic relatedness, phylogroup, host source, and major VF and ARG repertoires of these human-associated STs are summarized in Figure 5.

## Discussion

### To what extent are wild melting pots a legacy of human impact?

Among the JRBP animal community, host-associated *E. coli* carried a vast repertoire of virulence-associated genes; nearly half were known pathogens to humans. Additionally, numerous STs with strong human associations were present. These reached higher prevalence in the domestic animals, indicating their guts may experience higher levels of human impact, but wild animals were not unexposed. Genes encoding clinically relevant AMR were also present in both wild and domestic-sourced isolates, but resistance was higher among domestic samples. Approximately one-third of distinct ARGs were shared between wild and domestic animals, suggesting that domestic animals may provide a source of either continuous selective pressure (via antibiotics in their excrement) or inoculation (by ARG-containing bacteria), thus contributing to the detection of these ARGs in wildlife. Most of the geographic locations of the scats carrying ARGs were concentrated along the horse trail on the northern border of the preserve and on the eastern side where the cattle and goats were present (Figure S4). Other scats with high ARG prevalence were picked up along the most heavily trafficked foot trail circling Searsville Reservoir. The streams flowing into the preserve, the reservoir, and its drainage, could all be sources of ARGs, as these waters travel through agricultural areas and residential areas that use septic tanks, potentially acquiring various forms of environmental pollution such as agrichemicals and human and domestic animal waste before entering the preserve. The animals on the preserve appear to be differentially impacted by such environmental pollution, as no ARGs were identified in any deer or puma, despite some of the largest scat sample sizes from these species. And too, no resistance was detected in any small mammals, though these sample sizes were smaller. Conversely, 6 of 10 coyotes carried MDR *E. coli.* One probable explanation for this observation is their tendency for coprophagy, especially as most of these scat samples were collected along the horse trail, and 3 of 7 horse scat samples also contained MDR *E. coli*. It could also be explained by the higher propensity for coyotes to enter residential areas, increasing their contact with the human environment.

Our results indicate that substantial genetic diversity is encompassed by wild animal-derived *E. coli*, even within a small geographic area. The fact that we identified 14 potential clonal pairs shared between individuals of this community, amid such diversity illuminates the role of transmission and rapid rate at which it is likely occurring within this system. Indeed, 2 of those were ExPEC strains shared between predator and prey and 1 crossed the WUI. In addition to identifying potential transmission events within this community, we also captured considerable within-scat diversity, indicating that within-host *E. coli* genetic diversity remains underappreciated in wild animals, but reinforcing the likelihood that the vertebrate gut may serve as a melting pot of novel genetic combinations that have the potential to heighten the risk of zoonotic spillback of novel pathogens.

### Gauging the implications: A grain of salt?

It is important to note that resistances detected in our study tended to be to primarily older antibiotic agents. Previous studies suggest that *E. coli* of animal origin frequently show resistances to older antimicrobial agents, including tetracyclines, phenicols, sulfonamides, trimethoprim, and fosfomycin, attributed to the longer time frame these drugs have had to reach higher prevalence in the environment.^48^ Natural microbial communities carry a diversity of genes conferring AMR that also serve other such functions as efflux, cell-to-cell signaling, biocontrol of symbionts, or resistances to other environmental toxins, like heavy metals; known as the “intrinsic resistome”.^34^ Indeed, there is natural variation in the intrinsic AMR levels among *E. coli* strains, and some level of resistance occurs even in the absence of known history of antibiotic exposure.^49^ However, high levels of resistance frequently and rapidly evolve in bacterial communities exposed to antibiotics, either by mutation or HGT of ARGs. Because resistance usually comes at a metabolic cost (strains that carry them typically have reduced fitness), it is usually purged in the absence of continued antibiotic selective pressure and/or persistent introduction of the resistance genes to the bacterial environment.^49^ Criteria for ARGs posing “high risk” to public health include gene mobility, host pathogenicity, and the enrichment of specific resistances in anthropogenically impacted environments compared to non/low-human-impacted areas.^50^ For instance, chromosomal mutations leading to intrinsic resistance to colistin (a last-resort antibiotic) were documented decades ago, but the low HGT potential for those genes limited their ability to spread and thus had minimal clinical impact.^51^ However, the plasmid-mediated and thus highly mobile colistin resistance gene, *mcr-1,* has already spread to 7 different pathogenic species in 31 countries^52^ and occurs at reportedly high prevalence in human and livestock feces.^53^^,^^54^

Pathogenesis in *E. coli* might be a by-product of its genomic plasticity*,* thus enabling adaptation to a broad range of environments. Dubbed “the coincidental hypothesis for VFs”, it suggests that “virulence genes” evolved and are selectively maintained to serve other purposes in the bacterium’s ecology, especially in commensalism, as many “virulence factors” play roles in colonization of the gut and defense against other intestinal organisms.^37^ However, of over 1,400 infectious organisms identified in a comprehensive literature review, an alarming 75% were of zoonotic origin,^55^ underscoring the value of monitoring wild-animal carriage of human pathogens in order to protect public health. Such methodology is advocated by the “One Health” approach, which recognizes that human health is tightly linked to the health of animals and ecosystems.^56^ An extensive 2018 literature review on the occurrence of STEC in wild animals identified just 79 studies to-date, only 10% of which investigated the prevalence of STEC in wild animals in conjunction with livestock and humans, leading to the conclusion that the research had majorly overlooked the “One Health” approach, thus undercutting our potential to understand transmission routes between these spheres.^57^

### Limitations of the study

It has been reported that humans host an average of 3.5 *E. coli* phylogroups and likely many more transiently over their lifetime.^58^^,^^59^ However, such an average has yet to be estimated for any non-human host. Here, even within a single phylogroup from a single scat, we sequenced multiple STs, indicating that substantial diversity exists below the phylogroup level within a single gut environment. It follows that we have also yet to establish an ideal sample size to capture the within-host diversity of *E. coli.* Even results from such studies in humans have varied in the proposed number of isolates required to exhaust new strain discovery and to what level of probability that sample size has of obtaining minor strains.^60^ In a previous study, we estimated the total phylogroup diversity at the host species level and found that it positively corresponded with host body mass and is likely also influenced by other factors such as host diet and proximity to human impacts.^5^ This suggests that the “optimal” sample size to obtain the within-host diversity of *E. coli* will likely vary greatly across the animal kingdom.

Though our sample size of 143 genomes was relatively small compared to some previous studies such as one on 1,294 *E coli* isolates from humans, poultry, wild animals, and water on the Australian continent, our PCAs on the pan-genome closely resembled one another, with the major phylogroups clustering similarly and their PC1 axis accounting for 23.6% of the variance compared to 24.5% in our analysis.^61^ This could suggest that we obtained a good representation of the breadth of functional diversity documented in *E. coli* within just 68 animal hosts living in a small nature preserve. However, it could also indicate an underlying shortcoming of the reference database, as a key limitation of studies like these lies in the lack of description of the functional roles corresponding to the genetic diversity within bacterial genomes. Even the well-known laboratory model organism, *E. coli* K-12, still has 35% of its genes lacking experimental evidence of function and another 5% only known as pseudo- or phantom genes.^62^ This dramatic gap in the functional database was recapitulated here, as a total of 46% of the pan-genome of JRBP *E. coli* was undescribed in the COG database. Determining the function of a gene is highly involved and requires substantial time and laboratory work,^63^ but the fact that we have yet to understand the functional and metabolic roles of nearly half of the genes in a well-studied laboratory strain hinders attempts to unravel the eco-evolutionary dynamics of *E. coli* in diverse ecosystems. For example, though pipelines to predict AMR via genetic analysis, like ResFinder, are typically highly accurate,^64^ genotypic and phenotypic observations do not always agree. Does observing a resistant phenotype on antibiotic-containing agar represent what would happen in the gut, or could the context of the environment dictate gene expression? Deciphering the functional role of genes would also retroactively inform current WGS studies that have generated ample genetic data, but which are not as informative as they could be considering the gaps in gene-function databases.

Furthermore, much remains unknown surrounding the ability of pathogenic *E. coli* strains that make humans sick to also sicken wild animals. This is partially due to the deficit of studies in wild animals, as well as the challenges of determining a host’s well-being at the time of collection, especially if collections are taken opportunistically. Avian pathogenic *E. coli* (APEC) is a well-known pathogen of birds,^65^ but has likely received more attention due to its major impacts on the poultry industry. Future research should endeavor to address the limitations of opportunistic sampling by adopting methods of catch-and-release and tracking individuals over time to obtain important health and lifestyle metrics. We should also strive to sample a greater diversity of hosts and individuals from each host species to paint a more thorough picture of the ecology and movement of *E. coli* within a wild animal network. Such work should simultaneously aim to expand the database of *E. coli* genomes from a larger diversity of wild hosts, especially in the vicinity of agricultural lands. Such improvements to monitoring wildlife gut microbial communities will support source tracking efforts during outbreaks, enhancing our ability to prevent contamination, thus avoiding economic losses from crop contamination and expenses associated with medical treatment. This will also inform the propensity for wild animals to spread pathogens, as highly symptomatic individuals would shed more pathogens into the environment, and those carrying a greater diversity of strains run a higher risk of facilitating novel pathogen emergence. It may also assist in removing false blame placed on wild animals, thus bolstering wildlife conservation efforts.

### Conclusions

The prevalence of pathogenic and AMR *E. coli* in the JRBP animal community suggests that they experience direct or indirect anthropogenic impacts, and the similarity of some of these strains to one another across host species suggests transmission may be happening *in situ*. The occurrence of human-associated STs indicates that the surrounding human population and its legacy of impact have influenced the gut microbiomes of wild animals. We know home ranges of many species studied here extend beyond the bounds of “protection” into heavily populated areas. Human-wildlife conflict does occur here, for example, puma have reportedly preyed on domestic dogs and encounters between coyotes and residents in the area are common. Although there are no sewage treatment facilities in the immediate vicinity, the large number of septic tanks and leach fields, horse paddocks and chicken coops, may impact wildlife on the preserve. JRBP is probably unexceptional in this, as what was found here likely represents to varying degrees the impact of human populations around the globe on wild animals and their microbiomes. We highlight how harmful *E. coli* could be a result of our actions (or inactions) that introduce our waste, antibiotics, and AMR bacteria into the environment, where the microbes may then further evolve within the guts of wild animals. Thus, we recommend several interventions that seek to decrease the distribution and rapid evolution of harmful *E. coli*. These include drastically reigning in antibiotic use, properly disposing of antibiotics and other human waste to decrease our impact on the environment, improving water sanitation methods to better remove drugs, ensuring that compost reaches and maintains proper temperature to kill pathogenic *E. coli*,^66^ increasing protected spaces for wild animals to exist away from humans, and constructing wildlife corridors to reduce human-wildlife encounters.

We promote using *E. coli* as a model for understanding bacterial ecology and evolution in wild animal populations. With genomic data, we can assess the rate of carriage and propagation of AMR and pathogenic strains in the wild, evaluate the risk for zoonotic disease emergence, and potentially predict spillover and spillback events. All of this has strong implications for global public health, especially in the Anthropocene. Life on the planet is now enmeshed, highly interconnected, and faced with unprecedented environmental change. Surging human-wildlife interactions, alarming rise of AMR globally, and increased pollution are exerting strong selective pressures on bacterial communities to develop and maintain resistance mechanisms to heavy metals, agrichemicals, and antibiotics. These genes are incorporating into bacterial genomes and rapidly disseminating in the environment with poorly described and likely detrimental effects on human, animal, and ecosystem health.

## STAR★Methods

### Key resources table

**Table:** 

REAGENT or RESOURCE	SOURCE	IDENTIFIER
**Bacterial and virus strains**		

*E. coli* isolates from diverse animal hosts	Lagerstrom and Hadly^5^	https://doi.org/10.1128/aem.00142-23
*E. coli* str. K-12 sub-strain MG1655	GenBank	GenBank: U00096.3
*E. coli* O157:H7 str. Sakai	GenBank	GenBank: BA000007.3
*Escherichia fergusonii*	GenBank	GenBank: GCA_008064875.1

**Critical commercial assays**		

DNeasy Blood & Tissue Kit	Qiagen	Cat#69506
DNA Prep kit	Illumina	Cat#20060059

**Deposited data**		

R working scripts	This paper	https://doi.org/10.5281/zenodo.8161576
Raw sequencing data	This paper	BioProject: PRJNA992418; SRA: SRR25183432 - SRR25183576; BioSample: SAMN36349139 - SAMN36349283
EnteroBase constructed genomes	This paper	EnteroBase v1.1.4 (Barcodes: Table S7)
*de novo* constructed genomes	This paper	GenBank: JAUKHW000000000 - JAUKNI000000000

**Software and algorithms**		

Trim Galore v0.6.10	Felix Krueger, The Babraham Institute	https://www.bioinformatics.babraham.ac.uk/projects/trim_galore/
SPAdes v3.15.3	Prjibelski et al.^67^	https://cab.spbu.ru/software/spades/
BBMap v38.96	Bushnell^68^	https://sourceforge.net/projects/bbmap/files/BBMap_38.96.tar.gz/download
QUAST v5.2.0	Gurevich et al.^69^	https://quast.sourceforge.net/download.html
BUSCO v5.4.3	Simão et al.^70^	https://busco.ezlab.org/
EnteroBase v1.1.4	Zhou et al.^71^	https://enterobase.warwick.ac.uk/species/index/ecoli
ClermonTyper	Beghain et al.^72^	http://clermontyping.iame-research.center/
VirulenceFinder 2.0 software v2.0.3	Center for Genomic Epidemiology	https://bitbucket.org/genomicepidemiology/virulencefinder/downloads/
ResFinder 4.1 software v2.0.0	Center for Genomic Epidemiology	https://bitbucket.org/genomicepidemiology/resfinder.git/src
PlasmidFinder 2.1 software v2.0.1	Center for Genomic Epidemiology	https://bitbucket.org/genomicepidemiology/plasmidfinder/downloads/?tab=tags
mlplasmids v2.1.0	Arredondo-Alonso et al.^73^	https://gitlab.com/sirarredondo/mlplasmids
MOB-suite v3.1.0	Robertson et al.^74^; Robertson et al.^75^	https://github.com/phac-nml/mob-suite/blob/master/README.md
R package ggsankey v0.0.99999	Sjorberg^76^	https://github.com/davidsjoberg/ggsankey
Prokka v1.14.6	Seemann^77^	https://software.cqls.oregonstate.edu/updates/prokka-1.14.6/
Roary v3.13.0	Page et al.^78^	https://sanger-pathogens.github.io/Roary/
MAFFT v7.487	Katoh and Standley^79^	https://mafft.cbrc.jp/alignment/software/
R package ggplot2	Wickham^80^	https://cran.r-project.org/web/packages/ggplot2/index.html
RECOPHY	Sakoparnig^81^	https://recophy.unibas.ch/recophy/
Bowtie2	Langmead and Salzberg^82^	http://bowtie-bio.sourceforge.net/bowtie2/index.shtml
PhyML 3.0	Guindon et al.^83^	http://www.atgc-montpellier.fr/phyml/
RAxML-NG v. 1.1	Kozlov et al.^84^	https://github.com/amkozlov/raxml-ng
iTOL v5	Letunic and Bork^85^	https://itol.embl.de/itol.cgi

### Resource availability

#### Lead contact

Further information and requests for resources should be directed to and will be fulfilled by the lead contact, Katherine Lagerstrom (klager@stanford.edu).

#### Materials availability

This study did not generate new unique materials.

#### Data and code availability

• Assembled genome data have been deposited at GenBank and are publicly available under BioProject PRJNA992418 as of the date of publication. Accession numbers are listed in the key resources table.
• Raw sequencing data have been deposited in the Sequence Read Archive (SRA) and are publicly available as of the date of publication. Accession numbers are listed in the key resources table.
• All original code for analyses has been deposited at Zenodo and is publicly available. DOI is listed in the key resources table.
• Any additional information required to reanalyze the data reported in this paper is available from the corresponding author upon request.

### Method details

#### Study location

Jasper Ridge Biological Preserve (JRBP) is a 1,183-acre (∼5 km^2^) area of land owned by Stanford University in the eastern foothills of the Santa Cruz Mountains in California, USA. It has an extensive history of human impact ranging from indigenous occupation for thousands of years before European colonization, followed by livestock grazing, logging, agriculture, and recreation. Recreational use intensified when the construction of Searsville Dam in 1892 impounded surface waters as Searsville Reservoir. The area was later closed to the public after its designation as a biological preserve in 1973. The preserve itself encompasses a diversity of habitat types and is home to both native and introduced wild animal species. It contains a total of 34 marked and maintained trails and a field station, used by permission only. Though the interior of the preserve is protected from substantial human impact today, equestrian trails on the northern and eastern parts of the preserve are used frequently. A ranch adjacent to the northeastern border and equestrian facilities on the southeastern and western borders bring into proximity horses and cows. The southern and western borders abut lightly populated residential areas, gardens, and vineyards, with native habitat corridors along stream drainages, connecting the preserve to the large open spaces in the higher elevation Santa Cruz Mountains.

#### Strain acquisition and DNA extraction

In a previous study, 1,756 *E coli* colonies were isolated from 161 opportunistically collected fecal samples belonging to 17 animal species at JRBP including 3 domestic mammals, 12 wild mammals, 1 wild bird and 1 wild reptile to assess *E. coli* phylogroup diversity at the host species level.^5^ A representative collection of 145 of these isolates from 68 fecal samples collected along foot trails in JRBP (Figure S4) were selected for whole genome sequencing (WGS). The host species of selected scat samples were: black-tailed deer (*Odocoileus hemionus*; n = 7), bobcat (*Lynx rufus*; n = 7), California vole (*Microtus californicus*; n = 1), coyote (*Canis latrans*; n = 10), dusky-footed woodrat (*Neotoma fuscipes*; n = 1), gray fox (*Urocyon cinereoargenteus*; n = 6), long-tailed weasel (*Mustela frenata*; n = 1), opossum (*Didelphis marsupialis*; n = 1), puma (*Puma concolor*; n = 9), rabbit (*Lagomorpha*; n = 1), turkey (*Meleagris gallopavo*; n = 4), western fence lizard (*Sceloporus occidentalis*; n = 1), domestic cow (*Bos taurus*; n = 5), domestic horse (*Equus caballus*; n = 7), and 3 multi-individual group samples: a bulk sample from a domestic goat herd (*Capra hircus*), 3 independent samples from the same ground squirrel midden (*Otospermophilus beecheyi*), and 3 independent samples from a bat roost known to house Mexican free-tailed bats (*Tadarida brasiliensis*) and big brown bats (*Eptesicus fuscus*). The total *E. coli* isolate contribution was as follows: black-tailed deer (n = 18), bobcat (n = 14), California vole (n = 1), coyote (n = 23), dusky-footed woodrat (n = 2), gray fox (n = 13), long-tailed weasel (n = 2), opossum (n = 7), puma (n = 13), rabbit (n = 3), turkey (n = 10), western fence lizard (n = 2), domestic cow (n = 7), domestic horse (n = 16), goat herd (n = 6), squirrel midden (n = 5), and bat roost (n = 3). These data are summarized in Figure S6 and detailed in Table S3.

Isolates were incubated overnight at 37°C in 1mL Luria Broth (Difco, Sparks, Maryland), cells were pelleted out of suspension and resuspended in 1mL phosphate-buffered saline. DNA was extracted with the DNeasy Blood & Tissue Kit (Qiagen, Hilden, Germany) following the manufacturer’s recommendations. Final eluted DNA concentrations were quantified by fluorometry and stored at −20°C until library preparation.

#### Sequencing and genome assembly

In total, 145 isolates were sequenced at Admera Health (South Plainfield, NJ) with Illumina HiSeq or NextSeq paired-end sequencing following library preparation with Illumina DNA Prep kit (previously called Nextera DNA Flex) using 1/5 reactions. Adaptive quality and adapter trimming were performed using Trim Galore v0.6.10 (Felix Krueger, The Babraham Institute) with the --paired tag, and *de novo* genome assembly with SPAdes v3.15.3^67^ on paired-end reads and the --isolate tag. Post-processing was done with BBTools program BBMap v38.96^68^ to remove contigs shorter than 1000 bp. Genome assembly quality was assessed with QUAST v5.2.0^69^ with the “--conserved-genes-finding” tag which uses BUSCO v5.4.3.^70^ Genomes with N50 < 10,000 and/or a complete BUSCO score < 95% were removed for this study (n = 2). The average genome size for the remaining 143 genomes was 4,976,680 bp with coverage ranging from 19x – 681x and averaging 228x. The average complete BUSCO score was 98.57%.

Raw paired-end reads were also uploaded in EnteroBase v1.1.4^71^ (https://enterobase.warwick.ac.uk/species/index/ecoli), which runs an automated genome assembly pipeline and QC process. Assemblies were assigned to a sequence type (ST) based on the Achtman 7 gene MLST scheme, their *fimH* types were predicted by fimTyper, as well as O:H serotypes.^86^ Previous PCR-based phylogroup assignments were confirmed with the command-line version of ClermonTyper (http://clermontyping.iame-research.center/).^72^ GenBank accession numbers and EnteroBase barcodes are available in Table S7.

#### Identification of antimicrobial resistance genes, virulence factors, and plasmids

All whole genome assemblies were screened for virulence factors (VFs) relevant to *E. coli* using VirulenceFinder 2.0 software v2.0.3^87^^,^^88^ with method = blastn and search parameters set to 90% threshold for identity and 60% minimum coverage. Isolates were classified as ExPEC if positive for ≥ 2 of 5 key factors: *papA* and/or *papC* (P fimbriae), *sfa-focDE* (S and F1C fimbriae), *afa-draBC* (Dr-binding adhesins), *iutA* (aerobactin siderophore system), and *kpsMII* (group 2 capsules), and as UPEC if positive for ≥ 3 of 4 key factors: *chuA* (heme uptake), *fyuA* (yersiniabactin siderophore system), *vat* (vacuolating toxin), and *yfcV* (adhesin).^88^^,^^89^^,^^90^ Following previous methods,^88^ strains were considered positive for *afa*-*draBC* if both *afaB* and *afaC* were identified, and positive for *sfa*-*focDE* if a combination of *focC* or *sfaE* and also *focI* or *sfaD* were identified. Isolates that carried fewer than the necessary number of key UPEC or ExPEC VFs were assessed for the presence of 38 other major VFs deemed more impactful based on previous methods^46^^,^^88^ and included; *astA, cba, cea, cia, cib, clbB, cnf, cvaC, ehxA, espI, etsC, fimH, focG, hlyF, hra, ibeA, iha, ireA, iroN, irp2, iss, iucC, kpsE, lpfA, mcbA, mchB, mcmA, neuC, ompT, pic, sat, sfaS, sitA, senB, tcpC, terC, traT,* and *usp.* Strains that carried 5 or more of these major VFs were classified as “ExPEC-potential.” Strains that carried fewer than 5 of these but 10 or more total distinct VFs were classified as “ExPEC-like,” and strains carrying fewer than 10 total VFs were considered “low pathogenicity”, following previous methods,^46^ but increasing the minimum number of VFs to account for the since-expanded database.

ResFinder 4.1 software v2.0.0^64^^,^^91^^,^^92^ was used to identify acquired AMR genes and point mutations in all whole genome assemblies with species = “*Escherichia coli*” and search parameters set to 90% threshold and 60% minimum coverage.

The genomic contexts of contigs carrying resistance genes (chromosome versus plasmid) were predicted with mlplasmids v2.1.0^73^ and MOB-suite v3.1.0^74^^,^^75^ using their default parameters. In cases where the programs disagreed on a call, if the probability of the mlplasmids call was < 75%, the MOB-suite site prediction was used. The consensus of these results was used to create the alluvial diagram using R package ggsankey v0.0.99999.^76^ PlasmidFinder 2.1 software v2.0.1^91^^,^^93^ with search parameters set to 95% threshold for identity and 60% minimum coverage was also run on all genomes for comparison. PlasmidFinder results are reported in Table S3.

### Quantification and statistical analysis

Gene annotations were performed with Prokka v1.14.6.^77^ Pan-genome analyses were performed using Prokka annotations in Roary v3.13.0^78^ to predict core (shared by all or most strains) and accessory genes with the *-e* parameter for core-gene alignment by MAFFT v7.487.^79^ Principal component analyses (PCAs) were conducted using the ‘prcomp’ function in the *stats* package on the count matrix of all assigned genes, COG or EC numbers annotated by Prokka in the pan-genome. Ellipses for the PCA of ST10 isolates were calculated with the ‘stat_ellipses’ function in R package ggplot2.^80^

Assembled contig files were uploaded to the browser-based version of RECOPHY^81^ (https://recophy.unibas.ch/recophy/) which infers phylogenetic trees from WGS data by mapping each submitted sequence to each of the user-supplied references via Bowtie2.^82^ Then each of the individual reference alignments were merged to increase the quality of the inferred phylogeny. From these alignments, multiple sequence alignments were reconstructed to infer phylogenetic trees via PhyML 3.0^83^ with the default settings and general time-reversible (GTR) model. The reference genomes were *E. coli* str. K-12 sub-strain MG1655 (GenBank: U00096.3), *E. coli* O157:H7 str. Sakai (GenBank: BA000007.3), and *Escherichia fergusonii* (GenBank: GCA_008064875.1). A maximum-likelihood tree on the core gene alignment (Roary) of the human-associated STs was based on RAxML-NG v. 1.1^84^ under GTR+GAMMA with default parameters. The resulting trees were visualized and annotated in iTOL v5.^85^

Potential clonal relationships were defined as pairs of genomes with both ≤ 10 clonal SNPs and ≤ 10 recombined SNPs in the mix model matrix output by RECOPHY.^81^ Mutation rates in *E. coli* have been reported from 0.2 × 10^−10^ to 5 × 10^−10^ nucleotides/generation.^94^^,^^95^^,^^96^ Its high mutation rate suggests that there is an extremely small likelihood of near-perfect genome sharing unless derived from an identical source. Though clonal stability within *E. coli* STs has only been described to a limited extent,^47^ the criteria we used to identify potential strain-sharing events of ≤ 10 SNPs is comparable to the 9 SNP differences observed in a recurrent outbreak of ST10 clones in a broiler unit^97^ and the ≤ 6 SNP differences observed in within-host intra-clonal *E. coli* isolates from human UTIs and feces.^98^ We also report the cut-off of ≤ 100 SNPs often seen in the literature.^99^

## Supplemental information

Document S1. Figures S1–S6 and Tables S4 and S5

Table S1. Within-host *E. coli* diversity The first 3 columns list the sample IDs, host species, and number of isolates per scat sample for those which multiple *E. coli* isolates were sequenced. For each set of isolates from the same scat, the table details the number of distinct phylogroups (n_PGs) and the number of distinct sequence types (n_STs) those isolates contained. Also summarized are the number of clonal pairs (n_clonal_pairs) defined as 0 SNPs between isolates from the same scat, and the phylogroup from which multiple STs were isolated from the same scat (PG_with_diff_STs) where present.

Table S2. SNP analyses results Isolate metadata for all pairs of isolates with clonal and recombined SNP values of ≤ 100. The column entitled “sharing_type” describes whether the pair of isolates came from the same host species (intra-species), different host species (inter-species), or the same scat sample (within-sample). For those pairs shared between host species, the ST, serotype, pathotype, and AMR status are detailed.

Table S3. *E. coli* metadata This table contains the metadata for all 143 *E coli* isolates sequenced in this study. For each isolate, we report the host species, host status (wild or domestic), collection date and GPS location of the scat sample from which it derived. The estimated coverage of the *de novo* assembled genome and the total genome length was predicted in Enterobase. *E. coli* phylogroup, ST, whether the ST was considered human-associated (Y/N), serotype, fimH_type, ResFinder results, multi-drug resistance (MDR) defined as > 2 ARGs (Y/N), VirulenceFinder results, pathotype classification scores and pathotypes(s), and PlasmidFinder results as also reported.

Table S6. AMR Sankey diagram metadata For all 72 ARGs detected among 143 *E coli* genomes from JRBP, we report the isolate_ID, contig name carrying the ARG, the resistance gene name, percent identity, alignment length, coverage, position in reference and contig, phenotype, accession number, and genomic location prediction results from mlplasmids (Prob_Chromosome, Prob_Plasmid, Prediction) and MOB-suite (molecule_type, rep_type), and in cases where the two did not agree, the final call used to create the Sankey diagram for Figure 3.

Table S7. Accession numbers for genomic data For each genome sequenced in this study, identified by its isolate ID, the table lists the associated Enterobase barcode, Sequence Read Archive (SRA) accession number for the raw sequencing data, BioSample accession code, and GenBank accession code for the assembled genomes.
