# Supplementary material for: Impressive pan-genomic diversity of E. coli from a wild animal community near urban development reflects human impacts
Source: iScience. 2024 Feb 1;27(3):109072. doi: 10.1016/j.isci.2024.109072 (PMC10875580; doi:10.1016/j.isci.2024.109072)
Supplement: Document S1. Figures S1–S6 and Tables S4 and S5 [file mmc1.pdf]

iScience, Volume *27*

Supplemental information

Impressive pan-genomic diversity of *E. coli* from a wild animal community near urban development reflects human impacts

Katherine M. Lagerstrom, Nicholas C. Scales, and Elizabeth A. Hadly

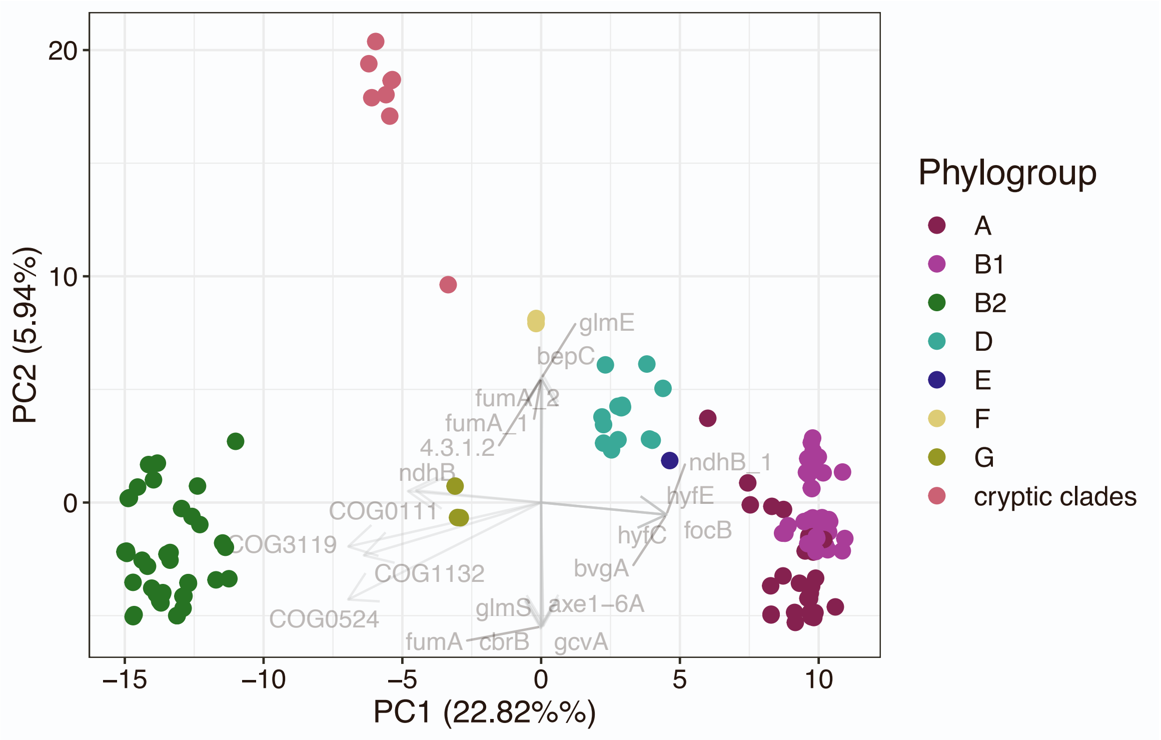

Figure S1. *Escherichia* functional similarity including isolates from the cryptic clades. Principal component analysis (PCA) of the entire pan-genome of isolates in this study (*n* = 143), shown colored by phylogroup association. Each dot corresponds to a genome in the first 2 principal components (PC). Vectors show the top 5 annotations with the largest PC1 and PC2 values in both positive and negative directions.

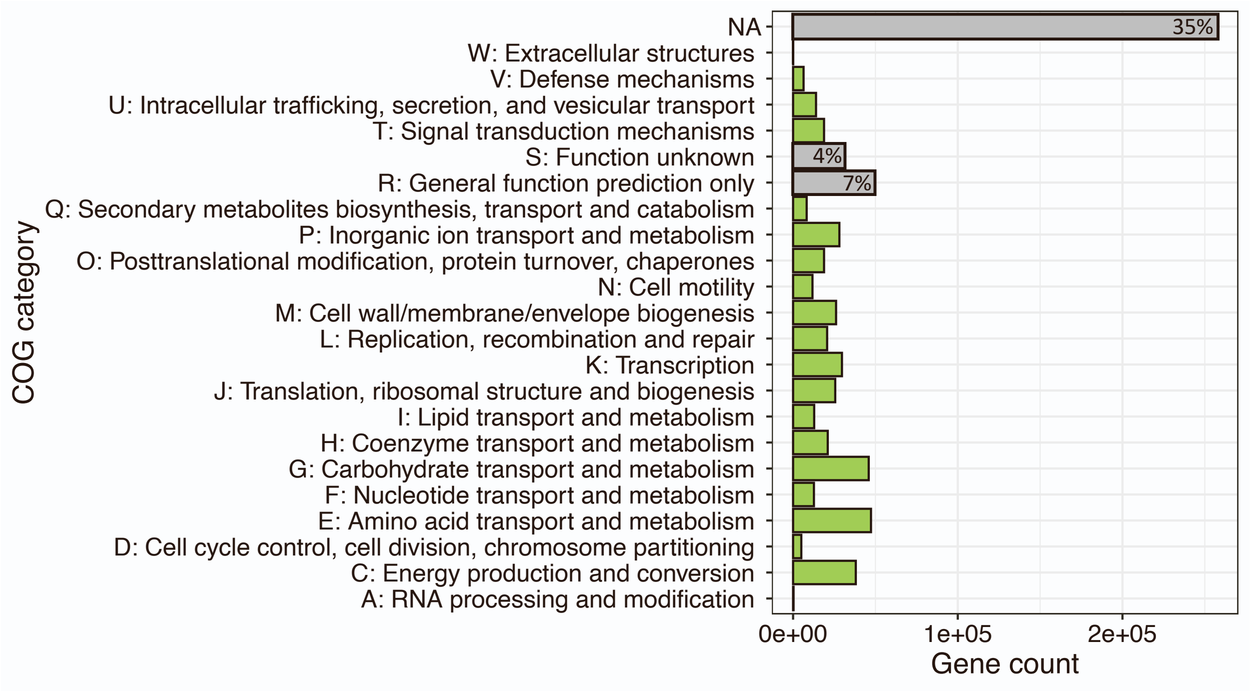

Figure S2. COG categories represented by JRBP *E. coli* pan-genome. Many annotations encompassed by JRBP *E. coli* pan-genome had unknown functions, with 46% assigned to COG categories of either 'NA', 'S: Function unknown', or 'R: General function prediction only'.

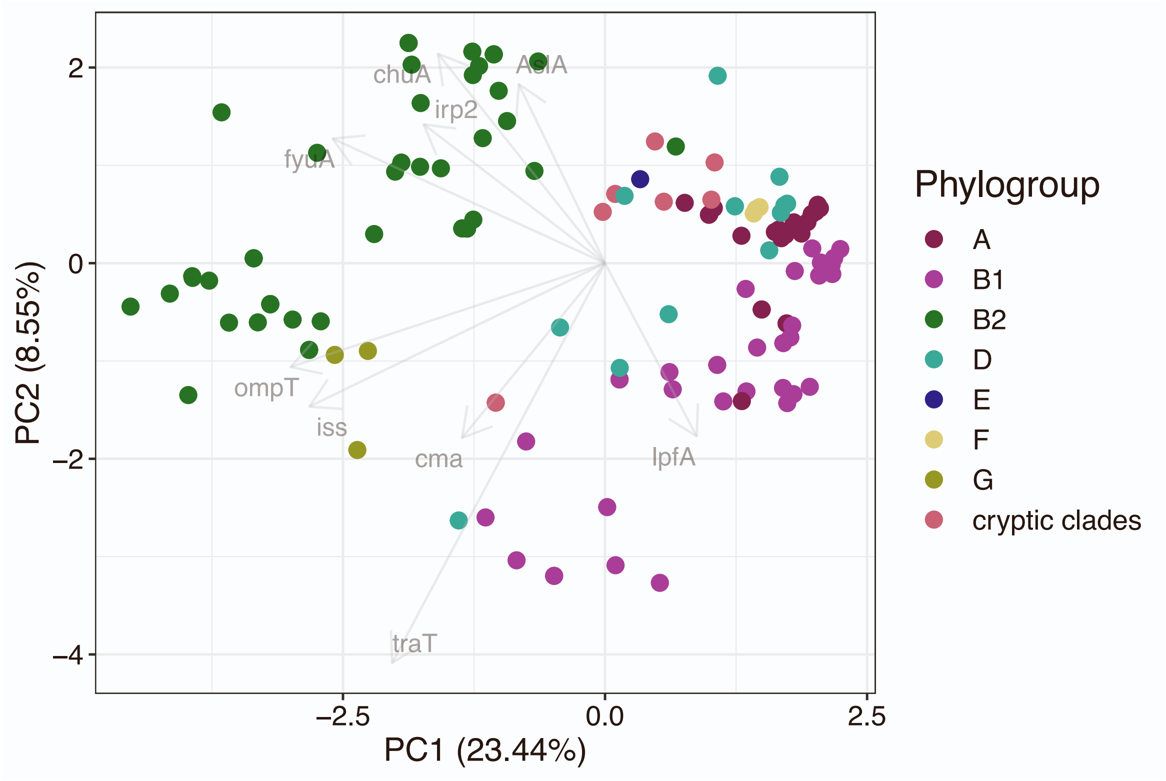

Figure S3. Virulence factor repertoire similarity. Principal component analysis (PCA) of the virulence factor (VF) repertoires of 143 genomes colored by phylogroup. Vectors show the top 5 VFs with the largest PC1 and PC2 values in both positive and negative directions.

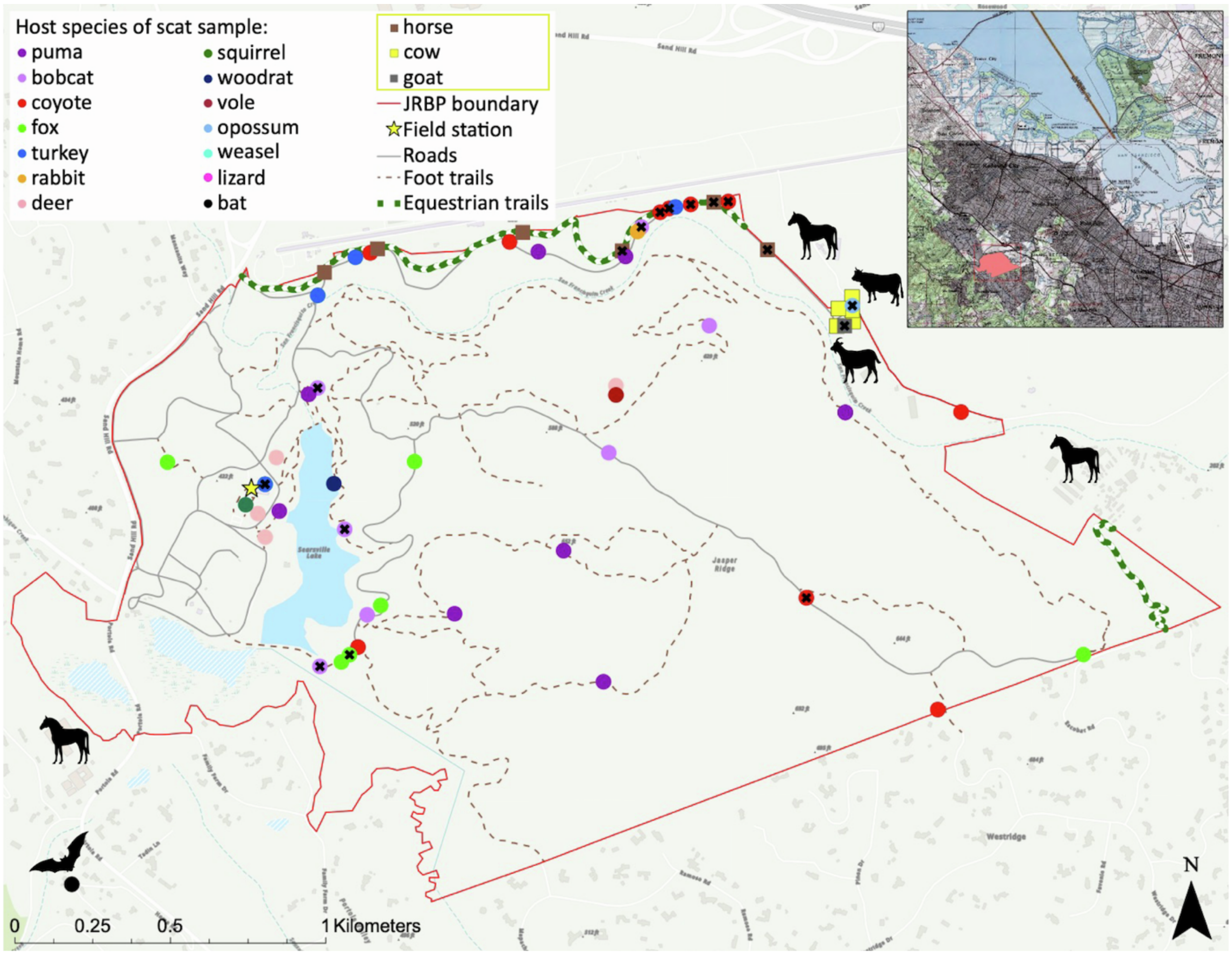

Figure S4. Study site and scat sample collection points. Locations of original scat samples the *E. coli* isolates sequenced here were recovered from at JRBP, California, USA. Samples marked with an "X" contained an *E. coli* isolate with one or more ARG. The bat silhouette marks the location of a barn on private property where a mixed species bat roost was sampled. Horse, goat, and cow silhouettes indicate where domestic animals are housed proximal to the preserve. The map inset shows the location of JRBP (red) imbedded in a densely populated area adjacent to San Francisco Bay, CA (ArcGIS Pro 3.0).

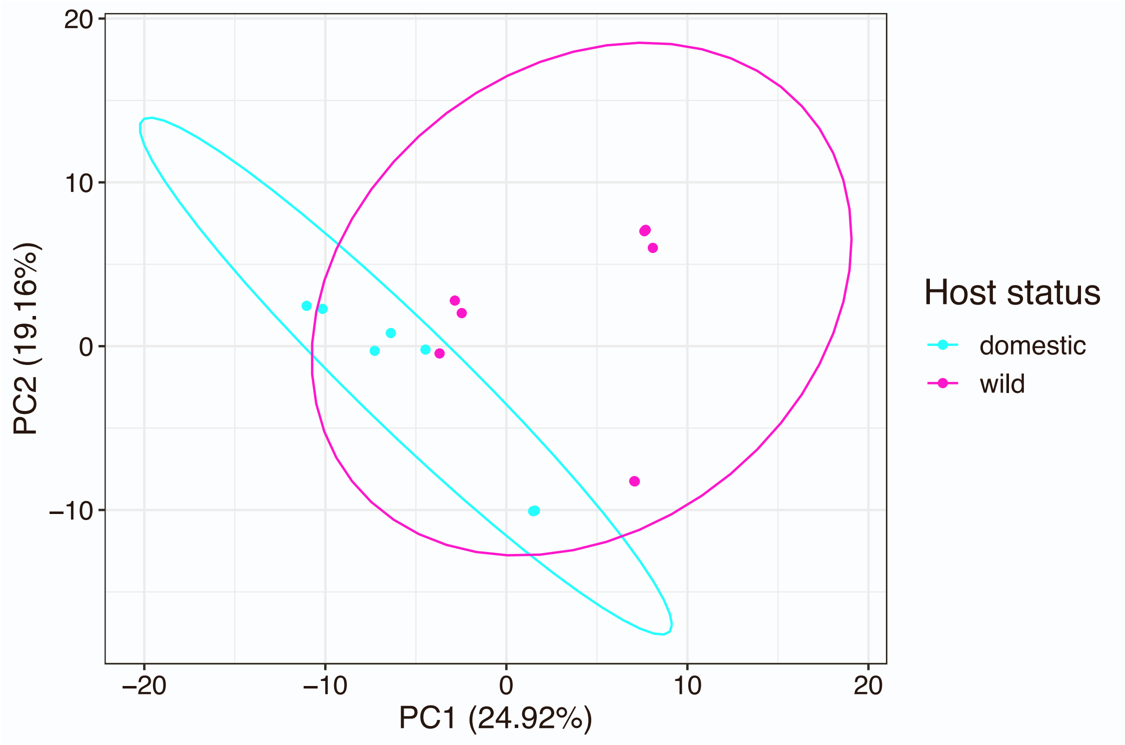

Figure S5. ST10 functional similarity between *E. coli* isolates from wild and domestic hosts. Principal component analysis (PCA) of *E. coli* belonging to ST10 from this study (domestic; *n* = 7, wild; *n* = 9). Each dot corresponds to a genome in the first 2 principal components (PC). Normal data ellipses were computed with the 'stat_ellipse' function in ggplot2.

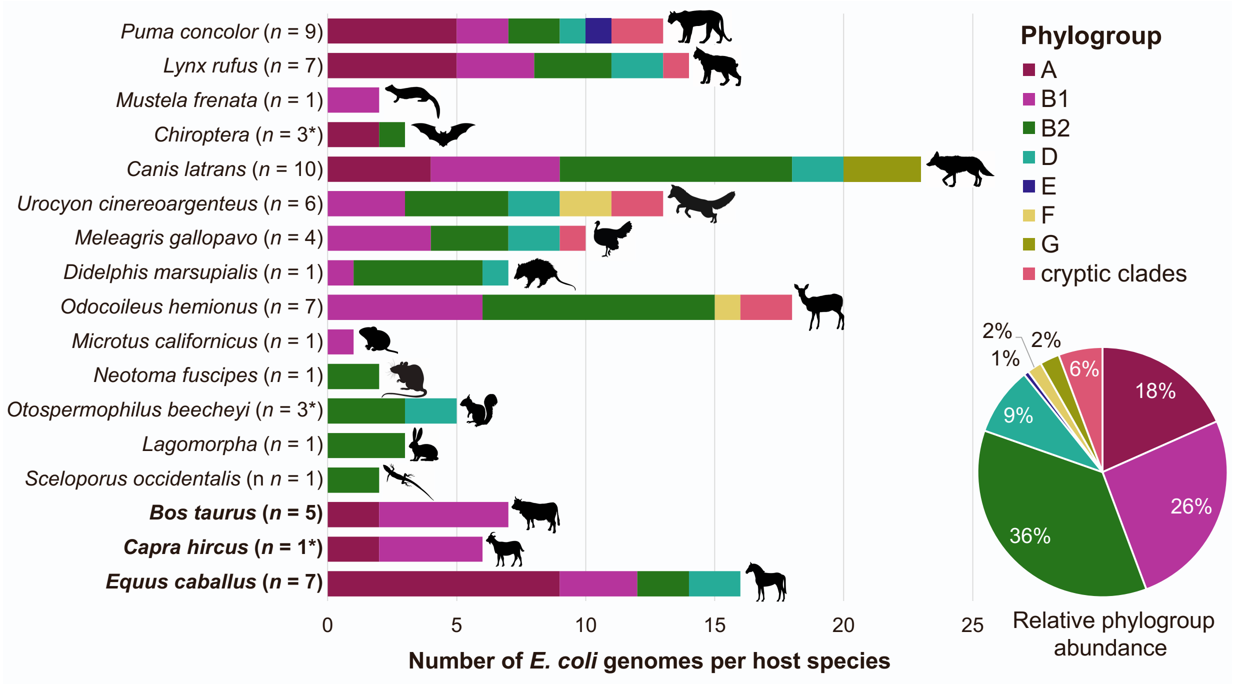

Figure S6. *E. coli* isolate contributions from animals at JRBP. For each host species on the y-axis, *n* denotes the number of scat samples per host and an asterisk indicates multi-individual group samples. Domestic host species are listed in bold. The pie chart illustrates the relative abundance of each phylogroup out of 145 total isolates sequenced in this study.

Table S4. Pathogenic *E. coli* assessment. Pathotype assignment criteria and prevalence of *E. coli* pathotypes among the JRBP isolates. *One UPEC was also aEPEC.

**Assignment criteria**	**JRBP *E. coli* (n=143)**		
**Extra-intestinal**	**Pathotype**	**n**	**Prevalence (%)**
≥ 2 key ExPEC VFs	ExPEC/non-UPEC	11	7.7
≥ 3 key UPEC VFs	UPEC/non-ExPEC	35	24.5
Meet both criteria above	ExPEC/UPEC	13	9.1
< 2 key, but > 5 major	ExPEC-potential	37	25.9
< 5 major but ≥ 10 total	ExPEC-like	47	32.9
< 10 total VFs	low pathogenicity	0	0
**Intra-intestinal**			
eae gene present (no *bfp*)	aEPEC	4*	2.8
stx1/*stx2* genes present (cytotoxin)	STEC	3	2.1

Table S5. Antimicrobial resistance and pathogen prevalence in *E. coli* at JRBP. The prevalence of AMR, MDR, ExPEC (including UPEC) and InPEC (STEC and aEPEC) among all *E. coli* isolates included in this study, between wild and domestic hosts, and across phylogroups, displayed as the count of genomes and the percent of total in parentheses.

Group	*n*	AMR occurrence (%)	MDR occurrence (%)	ExPEC+UPEC occurrence (%)	InPEC occurrence (%)
All genomes	143	23 (16.1)	12 (8.4)	59 (41.3)	7 (4.9)
**Host status**					
Wild	115	15 (13.0)	9 (7.8)	57 (49.6)	5 (4.3)
Domestic	28	8 (28.6)	3 (10.7)	2 (7.1)	2 (7.1)
**Phylogroup**					
A	29	6 (20.7)	3 (10.3)	0 (0.0)	1 (3.4)
B1	38	13 (34.2)	6 (15.8)	0 (0.0)	3 (7.9)
B2	47	1 (2.1)	0 (0.0)	46 (97.9)	1 (2.1)
D	14	1 (7.1)	1 (7.1)	7 (50.0)	2 (14.3)
E	1	0 (0.0)	0 (0.0)	0 (0.0)	0 (0.0)
F	3	0 (0.0)	0 (0.0)	0 (0.0)	0 (0.0)
G	3	2 (66.7)	2 (66.7)	2 (66.7)	0 (0.0)
Cryptic clades	8	0 (0.0)	0 (0.0)	4 (50.0)	0 (0.0)
